# Post-treatment changes in bowel and urinary function in prostate cancer patients treated with moderate or ultra-hypofractionation: A prospective cohort study

**DOI:** 10.1016/j.ctro.2025.100955

**Published:** 2025-04-07

**Authors:** W.D. Heemsbergen, F. Sinzabakira, K.C. de Vries, M. Franckena, M.E.M.C. Christianen, F.E. Froklage, H. Westerveld, L. Incrocci

**Affiliations:** Department of Radiotherapy, Erasmus MC Cancer Institute, University Medical Center Rotterdam, Dr Molewaterplein 40, 3015 GD Rotterdam, the Netherlands

**Keywords:** Prostate cancer, Hypofractionation, Radiotherapy, EPIC, EQ5D5L, Health-related quality of life

## Abstract

**Objective/Purpose:**

Hypofractionation (HF) has been established as safe in prostate cancer (PCa) trial populations. We evaluated post-treatment changes in health-related quality of life (HRQoL) outcomes after moderate (MHF) or ultra-hypofractionated (UHF) radiotherapy in a real-world PCa patient population.

**Methods:**

In this prospective cohort study, T1-4N0M0 PCa patients receiving MHF (20x3/3.1 Gy, n = 140) or UHF (7x6.1 Gy, n = 138) were assessed. UHF was not prescribed in case of T3b/T4 disease or an impaired baseline urinary function. Patients completed the EPIC urinary and bowel domains and the EQ-5D-5L (general HRQoL) at baseline and 6 months post-treatment. Thresholds for minimal clinically important differences (MCID) were defined at 8 %. Prognostic factors were assessed using regression models.

**Results:**

Proportion of the total cohort with MCID deterioration for bowel and urinary function was 20 % and 17 %, respectively. Deteriorations in bladder and bowel function significantly correlated with declined scores for general health, usual activities, pain/discomfort, and depression/anxiety. Predictive factors for urinary function deterioration at multivariable analysis were androgen deprivation therapy, age ≥ 75 year, MHF, and grade ≥ 2 acute urinary toxicity. For bowel function deterioration, no factors were identified.

**Conclusion:**

We assessed post-treatment changes in HRQoL in a clinical PCa patient population treated with MHF and UHF, and observed changes comparable to previous trial reports. Deteriorations in bowel and urinary function correlated with worse general health scores. Results for urinary function loss suggested a consequential effect of acute urinary toxicity.

## Highlights

• Moderate (MHF) and ultra-hypofractionation (UHF) is clinically implemented for prostate cancer.
• Post-treatment health-related quality of life was assessed in a real-world patient population.
• Hormonal therapy, MHF, and acute urinary toxicity associated with worsening in urinary domains.
• Deteriorations in bowel and urinary function correlated with worse general health scores.

## Introduction

In the past decade, hypofractionated radiotherapy (HFRT) for prostate cancer (PCa) has been established as a safe, effective, and patient-friendly treatment, with reduced costs and shortened treatment time [1]. Moderate hypofractionation (MHF), defined as 2.4–3.4 Gy per fraction, was evaluated in the CHHIP trial. The evaluated schedule of 60 Gy in daily fractions of 3 Gy was proven to be non-inferior in terms of late toxicity and tumour control compared to the conventional arm of 74 Gy in 2 Gy fractions, and has therefore been recommended for clinical practice by international guidelines [2,3]. Ultra-hypofractionation (UHF), defined as ≥ 5 Gy per fraction, was proven to be non-inferior with respect to tumour control and late toxicity in the HYPO-RT-PC randomized trial prescribing 42.7 Gy in fractions of 6.1 Gy, 3 times per week [4]. In the Pace-B UHF trial, 36.25 Gy was administered in fractions of 7.25 Gy (3–5 times per week, at centres discretion), demonstrating non-inferiority for clinical and biochemical tumour control [5]. In the CHHIP trial as well as the HYPO-RT-PC trial, acute toxicity was significantly higher in the HF arm compared to the control arm [3,4].

Patient-reported outcomes are valuable tools in oncology for assessing side effects and health-related quality of life (HRQoL) [[6], [7], [8]]. Literature on HRQoL data obtained from clinical MHF and UHF patient populations is currently limited. A commonly used and validated instrument for PCa is the Expanded Prostate Cancer Index Composite (EPIC) questionnaire with separate urinary, bowel, sexual, and hormonal domains, designed to measure patient function and bother [9]. For the CHHIP trial, results of the EPIC were presented with 5-year follow-up, showing no clinically relevant differences between the arms, confirming that MHF should be considered as standard of care [10]. With respect to post-treatment HRQoL levels after RT, several studies demonstrated that after the acute toxicity phase, HRQoL levels remain roughly stable from six months post-treatment onwards up to years thereafter [[11], [12], [13], [14]].

With the clinical introduction of HFRT at our institution, we started a prospective cohort study in 2019, collecting data on HRQoL and toxicity for PCa patients treated with curative intent (PRORAD study). We previously evaluated acute toxicity patterns for PRORAD patients who received HFRT [15]. In the current study, we assessed post-treatment changes in bowel and urinary EPIC scores, and its relation with baseline factors and simultaneous changes in general HRQoL aspects.

## Methods and Materials

### Study and patients

The “HRQoL in PCa patients treated with radiotherapy” (PRORAD) study is a prospective cohort study conducted at the Erasmus MC Cancer Institute that includes PCa patients receiving radiotherapy with curative intent according to various clinical protocols. Exclusion criteria of the PRORAD study were: previous pelvic radiotherapy, simultaneous treatment for other tumors, and not being able to fill out Dutch questionnaires. The study protocol was reviewed and approved by the medical ethics committee of Erasmus MC (MEC 2018–1711). The first patient was included in April 2019 and all participants provided written Informed Consent. The study protocol is registered at clinicaltrials.gov (NCT05645237). For the current study, we evaluated patients treated with MHF or UHF who filled out HRQoL questionnaires at baseline and at six months (M6) post-treatment.

### Diagnosis and treatment

Patients had histologically proven T1-4N0M0 PCa. A diagnostic magnetic resonance imaging (MRI) was available for 87 % (n = 242/278) of the patients (UHF 97 %, MHF 78 %). In 8/36 cases without MRI there was a contraindication (pacemaker, metal hip), of whom seven had a PSMA scan. In 28 patients it was decided that available imaging at the time was sufficient to proceed with RT planning without an additional MRI. For patients with high-risk disease characteristics, an additional PSMA scan was required (T3-T4, Gleason score ≥ 8 or 4 + 3, or PSA levels ≥ 20 ng/mL). HFRT was delivered using intensity-modulated radiation therapy (IMRT) and image-guided radiation therapy (IGRT) techniques, with prostate fiducial markers providing guidance. Treatment was delivered with 6 or 10 MV.

High-risk patients (T3-T4 or PSA˃20 ng/mL or Gleason 8–10) received androgen deprivation therapy (ADT) for 6 to 18 months, as prescribed by the treating urologist according to local protocols. MHF (60 Gy, 3 Gy daily fractions) was indicated for intermediate- to high-risk localized disease. In case of Gleason score ≥ 8 or cT3b, the fraction dose was 3.1 Gy (62 Gy in total) [3,16]. UHF (introduced in our clinic in 2020) was indicated for T1c-T3a disease requiring a diagnostic MRI showing no invasion of seminal vesicles. UHF (42 Gy, 6.1 Gy fractions) was delivered 3 times per week, including two weekends [4]. Part of the UHF patients (n = 35, 25 %) were treated within an adaptive online protocol (using the same margins to create the planning target volume) which was implemented during 2022. Applied margins were 0.7–0.8 cm, and the target volume was prostate only +/- (base) of the seminal vesicles (with no boost areas), depending on tumour characteristics: more details on target volume, treatment planning, and delivery were previously described [15].

### Health-related quality of life (HRQoL)

The urinary and bowel modules of the EPIC were administrated to score cancer-specific HRQoL [9]. Bowel function, bowel bother, urinary function, urinary bother, urinary incontinence, and urinary irritative/obstructive summary scores were calculated according to the EPIC scoring instructions. In general, individual items are linearly transformed to a 0–100 scale, with higher scores indicating better function or fewer problems [9]. The EPIC was scheduled before treatment and 6 months after the last RT fraction. Differences between M6 scores and baseline were calculated. Proposed Minimally clinically important difference (MCID) cut-off values for the EPIC in literature vary, and have been defined based on standard deviations as well as based on absolute differences [17,18]. Skolarus et al recommended MCIDs of 6 % to 9 % for urinary incontinence, 5 % to 7 % for urinary irritative symptoms, and 4 % to 6 % for bowel function, based on the short version of the EPIC (EPIC26) [17]. For the current study, we defined an 8 %-point cut-off as MCID for all calculated differences (declined ≤ -8%, unchanged −8% to + 8 %, improved ≥+8%). The general HRQoL was evaluated with the EQ-5D-5L, including a visual analogue scale (VAS) to indicate the perceived general health (with a score from 0 – worse, to 100 – best). The EQ-5D-5L is a European tool for assessing general HRQoL items mobility, self-care, usual activities, pain/discomfort, and anxiety/depression (scoring no, slight, moderate, severe, extreme problems) [19]. MCID difference for the VAS score was defined at 8 % as well.

### Statistical analysis

The statistical analysis was performed using IBM SPSS version 25 (IBM Corp, Armonk, New York). The Chi-square test was used to compare categorical and ordinal variables between UHF and MHF. Predictive factors for endpoints concerning MCID declines were evaluated in logistic regression models. We established a priori a list of potential predictive baseline factors based on literature and available data. In addition, we evaluated acute toxicity scores as predictive factor to evaluate potential consequential effects. Cutoff values (e.g. target volume > 60 cm^3^ vs < ) were determined post hoc based on actual distributions. To create a multivariable model, we performed backward conditional selection, starting with the significant factors at univariable analysis (with entrance and exclusion at 0.05 level). Calculated changes (M6-baseline) in EPIC domain scores and EQ-5D-5L dimension scores were correlated, using Spearman correlation. Significance level was set at p < 0.05 (two-sided testing).

## Results

### Baseline characteristics

**Fig. 1:**
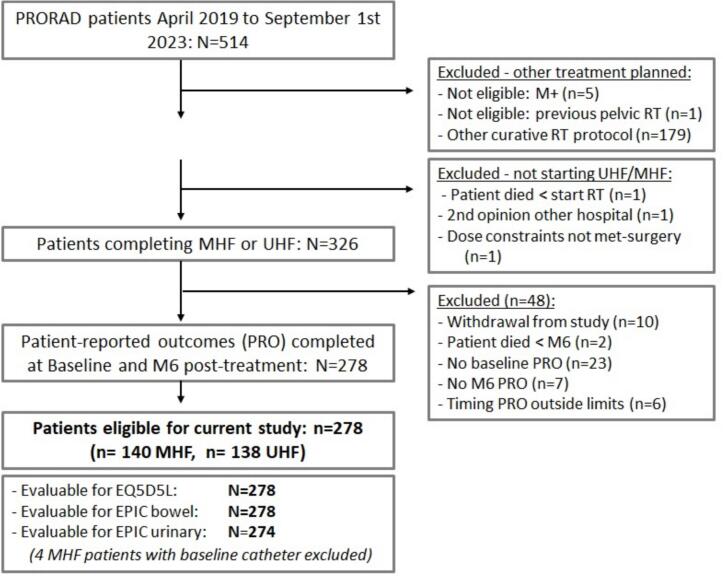
Flow chart describing the patient selection.

A total of 329 UHF and MHF patients were recruited from start of the PRORAD study up to September 2023 of whom 278 patients (n = 140 MHF, n = 138 UHF) fulfilled the selection criteria (Fig. 1, Table 1). The mean age at start radiotherapy was 73 year, and 47 % was ≥ 75 year. Since UHF was only administered in selected patients, more unfavourable tumour characteristics were present in the MHF group. The mean clinical target volume was larger for MHF as well (68 cm^3^ vs 58 cm^3^, p < 0.01). The distribution of comorbidity scores (indicated by the Charlson Comorbidity Index – CCI) was also unfavourable for MHF (Table 1). The average time interval between last RT fraction and M6 was 175 days. Four MHF patients had a transurethral catheter at baseline and were therefore not included in analyses concerning urinary endpoints (Fig. 1).

**Table 1:** Baseline patient, tumour, and treatment characteristics.

**Variables**	**MHF** N = 140	**UHF** N = 138	**p value**
Age at radiotherapy			
60–69 years*	21 %	25 %	0.4
70–74 years	29 %	33 %	
75–84 years	51 %	43 %	
Previous abdominal surgery	51 %	49 %	0.7
TURP/TURB	15 %	7.9 %	
Appendectomy	11 %	10 %	
Hernia inguinalis	9.5 %	12 %	
GI resection	5.0 %	3.6 %	
Kidney/stomach/gall bladder	8.6 %	12 %	
Hip surgery	6.4 %	3.6 %	
Current smoker	4.3 %	7.9 %	0.2
Diabetes	24 %	15 %	0.1
Cardiovascular history	32 %	26 %	0.2
CCI score (not age-adjusted)			0.02
0	41 %	49 %	
1	20 %	23 %	
2	18 %	20 %	
≥3	21 %	8 %	
T stage			<0.01
T1c-T2a	29 %	56 %	
T2b-T2c	18 %	26 %	
T3a	16 %	18 %	
T3b$	37 %	−	
Gleason score			<0.01
6–7	66 %	89 %	
8–10	34 %	11 %	
PSA (ng/mL)			<0.01
<10	39 %	52 %	
10–20	34 %	36 %	
>20	27 %	12 %	
Planned ADT			<0.01
None	42 %	81 %	
6 months	14 %	5 %	
1–3 years (mostly 18 months)	44 %	14 %	
LUTS medication	44 %	18 %	<0.01
Anticoagulants	39 %	35 %	0.5

*including 2 patients of 50 and 54 years old. $ including 4 patients with a T4 tumor.

Abbreviations: ADT = androgen deprivation therapy; CCI=Charlson Comorbidity Score; LUTS = lower urinary tract symptoms; MHF = Moderate hypofractionation; TURP/TURB = transurethral resection of the prostate/bladder; UHF = Ultra-hypofractionation.

### Distributions of calculated scores

**Fig. 2:**
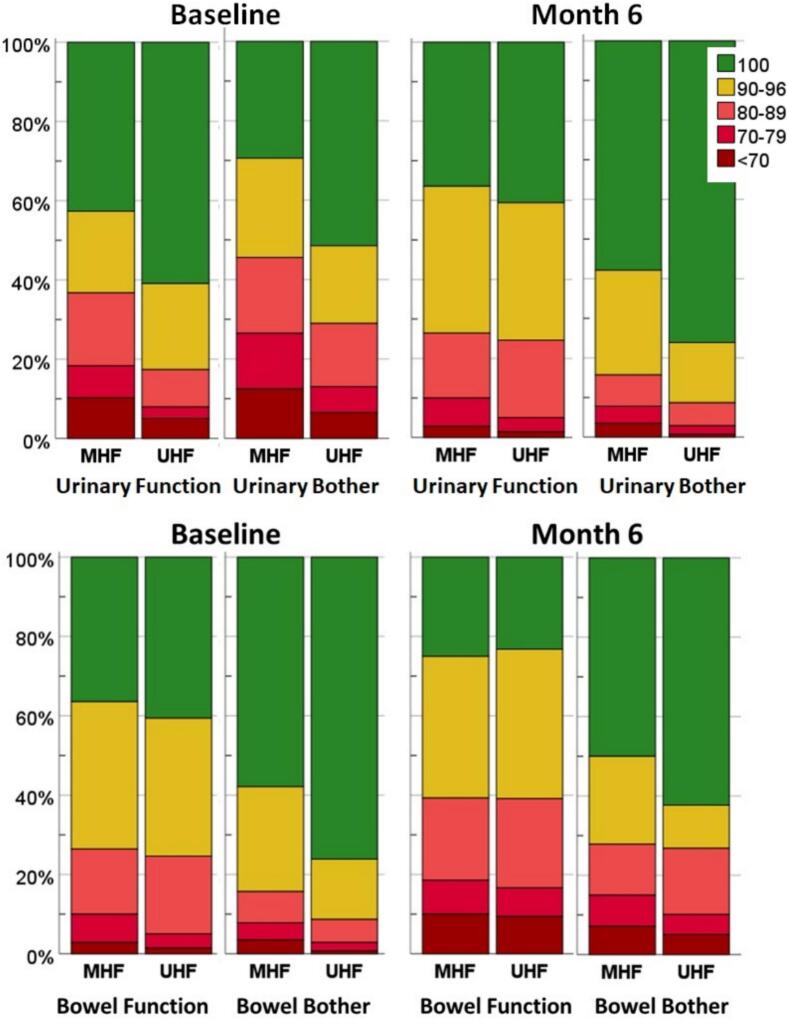
Distribution of Baseline scores + Month 6 (M6) scores for the EPIC domains bowel function & bother, and urinary function & bother. Green colour indicates the highest possible score of 100 (perfect function, no bother), darkest red indicate the worst group reporting worst function/bother (score < 70). Scores 97–99 are not possible in EPIC domain scores, therefore not included in the colour legend. (For interpretation of the references to colour in this figure legend, the reader is referred to the web version of this article.)

The calculated baseline EPIC scores for EPIC subdomains had a skewed distribution with the majority of patients scoring between 90–100. Baseline VAS health scores were mainly between 70–100. (Fig. 2, supplementary Fig. S1). Calculated differences in domain scores (M6-baseline) showed normal distributions (supplementary Fig. S1). Mean and median values are reported in supplementary Table S1, showing high mean values (range 86–98) and small mean differences (range −4.5, +2.2). With respect to changes in bowel function, most changes were negative changes, i.e. deteriorations, while for changes in urinary function and VAS health scores the distributions showed that both decreased and increased functioning were present (supplementary Fig. S1+S2).

### MHF HRQoL changes

MCID deteriorations with respect to bowel functioning and bowel bother were observed in 17 % and 21 %, respectively for MHF. Six percent experienced improvement (Table 2). Four MHF patients (with no catheter at baseline) had a catheter at M6, and they were scored as having a MCID deterioration for all urinary subdomains. Urinary subdomains showed MCID declines in 24 % (function), 20 % (bother), 29 % (incontinence), and 19 % (irritative-obstructive) of the MHF group. For all urinary subdomains, we also observed MCID improvements in 18 %–23 % of the patients (Table 2). In addition, we evaluated MCID distributions for urinary domains in the subgroup with a good baseline urinary function with scores between 90–100 (Supplementary Table S2). These results show similar percentages of MCID declines, and smaller groups with MCID improvements, except for the irritative-obstructive subdomain where 21 % still reports improvement. In supplementary Fig. S3 is depicted that patients with a MCID decline for bother, have in particular worse function scores for the sub-items of uncontrolled stools and leakage of urine, compared to patients without MCID bother decline. Worse MCID VAS health scores were reported by 24 % of the MHF patients while 18 % reported improvement (Table 2).

**Table 2:** Distribution of MCID changes (Month 6-Baseline) for EPIC subdomains and VAS health score, for the total group, MHF, and UHF.

**EPIC subdomain**	**Total group (N = 278)**	**MHF (N = 140)**	**UHF** **(N = 138)**	**p value** **MHF vs UHF**
**VAS health score**				
No MCID	58.6 %	57.9 %	59.4 %	0.6
Improved (≥+8%)	15.8 %	17.9 %	13.8 %	
Worsened (≤ −8%)	25.5 %	24.3 %	26.8 %	
**Bowel function**				
No MCID	72.7 %	76.4 %	68.8 %	0.4
Improved (≥+8%)	7.2 %	6.4 %	8.0 %	
Worsened (≤ −8%)	20.1 %	17.1 %	23.2 %	
**Bowel bother**				
No MCID	74.5 %	72.9 %	76.1 %	0.8
Improved (≥+8%)	5.8 %	6.4 %	5.1 %	
Worsened (≤ −8%)	19.8 %	20.7 %	18.8 %	
**Urinary function**				
No MCID	65.0 %	52.9 %	76.8 %	<0.001
Improved (≥+8%)	18.2 %	22.8 %	13.8 %	
Worsened (≤ −8%)	16.8 %	24.3 %	9.4 %	
**Urinary bother**				
No MCID	67.5 %	61.0 %	73.9 %	0.06
Improved (≥+8%)	16.8 %	19.1 %	14.5 %	
Worsened (≤ −8%)	15.7 %	19.9 %	11.6 %	
**Urinary incontinence**				
No MCID	64.2 %	53.7 %	74.6 %	0.001
Improved (≥+8%)	13.9 %	17.6 %	10.1 %	
Worsened (≤ −8%)	21.9 %	28.7 %	15.2 %	
**Urinary irritative-obstructive**				
No MCID	67.5 %	60.3 %	74.6 %	0.013
Improved (≥+8%)	19.0 %	20.6 %	17.4 %	
Worsened (≤ −8%)	13.5 %	19.1 %	8.0 %	

Abbreviations: EPIC = Expanded Prostate Index Composite; MHF = moderate hypofractionation; MCID = minimal clinically important differences; UHF = ultra hypofractionation; VAS = visual analogue scale.

### UHF HRQoL changes

In the UHF subcohort, 23 % reported a MCID decline in bowel function and 19 % a decline in bowel bother, while 8 % and 5 % reported improved function and bother, respectively (Table 2). Worse urinary functioning was reported in 9 % while 14 % reported improved functioning. Also for urinary bother (12 % decline, 15 % improvement) and irritative-obstructive (8 %, 17 %), more patients reported improvement. MCID urinary incontinence was reported in 15 % (10 % had improved scores). Comparing results within the UHF group for online replanning (n = 35) versus standard procedure (n = 103), showed no or small non-significant differences (results not shown). VAS health scores showed MCID declines in 27 % of the UHF patients, and 14 % reported improvement.

### EQ-5D-5L dimensions

The results for the EQ-5D-5L dimensions are summarized in Table 3. It shows that almost every patient had no problems at baseline with self-care, and the most frequent baseline problem concerned pain/discomfort. All dimensions, except anxiety/depression, had on average significant worse scores at M6. The most frequent MCID deteriorations were found for pain (25 %) and usual activities (18 %) (Table 3).

**Table 3:** Distribution of changes in EQ-5D-5L dimension (M6-Baseline), for total group and MHF/UHF subgroups. P values (MHF vs UHF) are given for distribution of baseline scores and for distribution of changes.

**EPIC subdomain**	**Total group (N = 278)**	**MHF (N = 140)**	**UHF** **(N = 138)**	**p value** **MHF vs UHF**
**Mobility**				
Baseline no problem#	77.0 %	71.4 %	82.6 %	0.04
M6 No change	72.3 %	70.7 %	73.9 %	0.2
M6 Improved	8.7 %	10.0 %	7.2 %	
M6 Worsened	19.0 %	19.3 %	19.0 %	
**Self-care**				
Baseline no problem#	97.5 %	97.9 %	97.1 %	0.5
M6 No change	95.0 %	95.0 %	94.9 %	0.6
M6 Improved	0.4 %	0 %	0.7 %	
M6 Worsened	4.7 %	5.0 %	4.3 %	
**Usual activities**				
Baseline no problem#	82.7 %	80.7 %	84.8 %	0.3
M6 No change	74.8 %	72.9 %	76.8 %	0.7
M6 Improved	7.6 %	7.9 %	7.2 %	
M6 Worsened	17.6 %	19.3 %	15.9 %	
**Pain/discomfort**				
Baseline no problem#	61.2 %	57.9 %	64.5 %	0.2
M6 No change	62.2 %	61.4 %	63.0 %	0.6
M6 Improved	13.3 %	14.3 %	12.3 %	
M6 Worsened	24.5 %	24.3 %	24.6 %	
**Anxiety/depression**				
Baseline no problem#	81.7 %	79.3 %	84.1 %	0.2
M6 No change	80.2 %	76.4 %	84.1 %	0.3
M6 Improved	11.1 %	12.9 %	9.4 %	
M6 Worsened	8.6 %	10.7 %	6.5 %	

# Remaining patients indicated mostly slight or moderate problems at baseline.

Abbreviations: EPIC = Expanded Prostate Index Composite; MHF = moderate hypofractionation; UHF = ultra hypofractionation.

### Differences between UHF and MHF

All EPIC subdomain scores showed significantly higher mean scores for UHF compared to MHF at baseline and M6, except for bowel function and bother (supplementary Table S1). The most significant differences were observed for the subdomain urinary incontinence (mean scores at baseline/M6 of 88/86 for MHF versus 95/94 for UHF). The baseline VAS Health score was on average 82 for UHF and 80 for MHF (p = 0.045) (supplementary Fig. S2). With respect to the calculated differences Baseline-M6, there were no significant differences observed between UHF and MHF (supplementary Table S1, Fig. S2). With respect to MCID distributions, in particular for urinary subdomains significant differences between UHF and MHF were observed (Table 3).

### Predictive factors for MCID deteriorations

For the endpoint of MCID decline in bowel function and bowel bother, none of the evaluated factors were predictive (Table 4). For bowel bother, acute grade ≥ 2 urinary toxicity (OR = 1.75, p = 0.065), and previous abdominal surgery (OR = 0.56, p = 0.059) were close to significance. For both urinary function and bother, T3 tumour (vs T1-2, OR = 2.7 and 2.0), ADT prescription (OR = 2.4 and 2.8), and acute grade ≥ 2 urinary toxicity (OR = 3.5 and 3.0) were significantly associated with increased risks. For urinary function, age ≥ 75 year (OR = 1.99, p = 0.04) and MHF treatment (OR = 3.1, p = 0.001) were associated with increased risks as well. Both baseline *bowel* function score (OR = 2.2, p = 0.04) and acute grade ≥ 2 *bowel* toxicity (OR = 2.1, p = 0.03) were predictive for MCID *urinary* bother but showed no significant associations with the urinary function endpoint (OR = 1.7 and OR = 1.2, respectively). At multivariable analysis, remaining factors for MCID urinary function decline were: ADT (OR = 2.2, 95 % CI 1.0–4.8), age ≥ 75 year (OR = 2.2, 95 % CI = 1.0–4.6), MHF (OR = 2.4, 95 % CI = 1.0–5.9), and grade ≥ 2 acute urinary toxicity (OR = 2.8, 95 % CI = 1.3–6.0). For MCID decline in VAS health, ADT was predictive (p = 0.05), with a relative risk of 1.7 for patients treated with ADT (Supplementary Table S3).

**Table 4:** Univariable logistic regression models for the endpoints of MCID deteriorations in bowel and urinary functioning (Odds Ratios with 95 % confidence intervals), N = 278 (N = 274 for urinary endpoints). Significant results are in bold.

	**Bowel function** **MCID ≤ -8%** **OR (95 % CI)**	**Bowel bother** **MCID ≤ -8%** **OR (95 % CI)**	**Urinary function MCID ≤ -8%** **OR (95 % CI)**	**Urinary bother** **MCID ≤ -8%** **OR (95 % CI)**
*Characteristics*				
T3 vs T1-2	1.26 (0.7–2.4)	1.33 (0.7–2.6)	**2.71** (1.4–5.4)	**1.99** (1.0–4.0)
ADT yes vs no	0.97 (0.5–1.8)	0.83 (0.4–1.5)	**2.34** (1.2–4.4)	**2.78** (1.4–5.4)
MHF vs UHF	0.69 (0.4–1.2)	1.13 (0.6–2.0)	**3.08** (1.5–6.2)	1.89 (0.97–3.7)
Age ≥ 75 vs < 75	1.08 (0.6–1.9)	1.03 (0.6--1.9)	**1.99** (1.1--3.8)	1.73 (0.9–3.3)
CCI score ≥ 2	1.05 (0.6–1.9)	0.71 (0.4–1.4)	1.70 (0.9–3.2)	1.39 (0.7–2.7)
Diabetes	0.88 (0.4–1.9)	1.77 (0.9–3.5)	1.39 (0.6–3.0)	1.13 (0.5–2.5)
Cardiovascular history	1.08 (0.6–2.0)	0.71 (0.4–1.4)	1.72 (0.9–3.3)	1.21 (0.6–1.4)
Abdominal surgery	0.93 (0.5–1.7)	0.56 (0.3–1.0)	1.11 (0.6–2.1)	1.31 (0.7–2.5)
LUTS medication	0.97 (0.5–1.8)	1.36 (0.7–2.5)	1.65 (0.9–3.2)	1.67 (0.9–3.3)
CTV > 60 cm^3^ vs <	1.50 (0.8–2.9)	1.09 (0.6–2.1)	1.95 (0.9–4.3)	1.87 (0.9–3.9)
*Baseline scores*				
VAS health < 80 vs ≥ 80	0.64 (0.3–1.2)	1.02 (0.5–1.9)	1.16 (0.6–2.2)	1.66 (0.8–3.2)
Bowel function < 90 vs ≥ 90	0.91 (0.4–2.0)	1.37 (0.6–2.9)	1.71 (0.8–3.7)	**2.25** (1.04–4.9)
Urinary function < 90 vs ≥ 90	1.10 (0.6–2.1)	1.27 (0.7–2.4)	1.08 (0.5–2.2)	0.92 (0.4–1.9)
*Acute toxicity#*				
Acute grade ≥ 2 Bowel	1.20 (0.7–2.2)	1.36 (0.7–2.5)	1.17 (0.6–2.2)	**2.10** (1.1–4.0)
Acute grade ≥ 2 Urinary	1.16 (0.6–2.1)	1.75 (1.0–3.2)	**3.47** (1.8–6.7)	**3.01** (1.5–5.9)

Abbreviations: ADT = androgen deprivation therapy; CCI=Charlson Comorbidity Index; CI = confidence interval; CTV = clinical target volume; MHF = moderate hypofractionation; LUTS = lower urinary tract symptoms; MCID = minimal clinically important differences; OR = odds ratio; UHF = ultra hypofractionation; VAS = visual analogue scale.

# acute toxicity scores were previously assigned, as reported by Sinzabakira et al [15].

$ available for 214 patients.

### Associations between EPIC changes and EQ-5D-5L changes

Changes (M6-baseline) in all EPIC subdomains except urinary functioning, showed significant correlations with simultaneous changes in one or more EQ-5D-5L dimensions, indicating declines in experienced health in case of deteriorated bowel and/or urinary function that bothered them (Supplementary Table S4). In particular changes in the EPIC subdomains bowel function, bowel bother, urinary bother, and urinary irritative showed significant correlations with changes in the general health items Usual activities, Pain/discomfort, and Anxiety/depression. The strongest associations were found between bowel function-usual activities (Spearman rank correlation 0.23, p < 0.001), bowel bother-usual activities (0.30, p < 0.001), bowel function/bother-anxiety/depression (0.18, p = 0.002), and urinary bother-usual activities (0.17, p = 0.005).

## Discussion

We evaluated early post-treatment changes between baseline and M6 in cancer-specific bowel and urinary HRQoL after HFRT for PCa in a clinical setting, in 278 men treated with MHF or UHF. Observed MCID proportions of worse scores for the urinary and bowel EPIC subdomains varied between 10 %-30 %. The most frequent and greatest deteriorations were observed for bowel function and bowel bother, which is in agreement with literature on radiotherapy effects on HRQoL [7,11,20]. Furthermore, deteriorations in bowel function showed strong correlations with reported declines in EQ-5D-5L dimensions, in particular with respect to problems with usual activities, anxiety/depression, and experienced overall health, suggesting a significant toxicity burden in these patients.

Since UHF was only prescribed in selected patients, the baseline profiles of urinary symptoms, LUTS medication, ADT prescription, and tumour characteristics were different between UHF and MHF. Since our database likely does not capture the whole clinical decision making process of the patient and physician when choosing UHF or MHF, our study objectives did not include direct comparisons between UHF and MHF outcomes, since they might not be valid even when adjusting for baseline factors at multivariable analysis.

It was remarkable that urinary bother scores were in general worse compared to urinary function scores. This reflects the fact that weak stream / incomplete emptying is a question for the urinary bother subdomain in the EPIC list (indicated as a problem by about 30 % of our study patients), but this item is not included in the EPIC subdomain of urinary functioning.

In our study, baseline bowel function score and acute grade ≥ 2 bowel toxicity were predictive for urinary bother worsening. This suggests complex mechanisms about development of symptoms and how patients perceive symptoms. In literature, correlations between bladder and intestinal function have been described previously. Underlying mechanisms have been proposed, like rectal filling inducing chronic pressure on the bladder causing over-activity of the bladder, and cross-organ sensitization between the bowel and bladder in patients with bowel disease [21].

We found baseline EPIC scores not to be predictive for MCID changes at M6, which was also observed in a previous study of Pinkawa et al in PCa patients treated with conventionally fractionated 3D conformal radiotherapy [22]. They also reported that scores at the end of RT were predictive for late EPIC scores. In our study, acute urinary toxicity grade ≥ 2 was predictive for post-treatment MCID declines in urinary function and bother scores, but this was not the case for bowel scores. In a previous study of Fiorino et al, evaluating conventional and HF radiotherapy in a postoperative setting, a consequential component for late urinary toxicity was suggested as well [23].

ADT was also associated with a decline in urinary function at M6 in our study. This was also observed in the study by Sanda et al, who reported on HRQoL at 6 and 24 months post-treatment (using the EPIC-26) among PCa survivors treated with brachytherapy or external beam RT [12]. They reported this impact at 6 months but not at 24 months, when ADT was stopped. ADT can improve urinary function in PCa patients with LUTS related to an enlarged prostate, but this effect was not measured in our study since most patients already had started ADT when they completed the baseline questionnaire. ADT may also increase irritative symptoms such as nocturia, as reported in several studies [12,24].

Many studies have explored how (conventional) radiotherapy and other treatment options affect HRQoL in PCa patients. Outcomes of such studies are essential input for shared decision making in clinical practice. With respect to the impact of HFRT protocols on HRQoL, only limited literature is currently available, and mainly for selected patients entered in clinical trials. Our study population contained a large group of patients aged > 75 years, and a large subgroup with multiple comorbidities, which deviates from patient profiles included in clinical trials. Hence, deviating (worse) outcomes with respect to HRQoL and toxicity might be a realistic scenario, since such a clinical patient population is more vulnerable. Wilson et al. investigated whether HRQoL profiles were different for patients in the CHHIP trial aged ≥ 75 years versus < 75 years, and found only a slightly increased risk for patient-reported bowel bother for older patients [25]. In the ProtecT study, age was not predictive for HRQoL scores, while for other treatment options (like prostatectomy) age was associated with worse outcomes [20].

Several studies showed that the impact of RT on toxicity and HRQoL levels at M6 post-treatment are indicative for HRQoL levels at 1–5 years of follow-up [[11], [12], [13], [14]]. For instance, in the CHHIPP trial an average reduction in the EPIC bowel function and bother score at M6 of about −5 was observed (in the same range as our results), which remained stable up to 24 months [13]. In the ProtecT trial, patients in the RT arm received about 7 weeks of RT within 9 months after randomization, and EPIC questionnaires were distributed after randomization at 6 months, 12 months, and annually thereafter [11]. Their measurement at 12 months post-randomization is therefore comparable to our M6 time point, showing stable measurements compared to their measurements thereafter up to 72 months, for most EPIC bowel and urinary endpoints. With respect to bloody stools, their results show some worsening in scores over time, and faecal incontinence and loose stools show some improvement [11]. Hoffman et al reported stable EPIC domain scores for the RT group in the period of 6 months after treatment initiation up to 60 months, in a prospective cohort study [14]. However, for a comprehensive evaluation of late toxicity, including RT effects like rectal bleeding and urinary obstruction, extended follow-up and data collection is required. For the current HF cohort, we expect to have mature results for a sufficient sample size in 2027. With prolonged follow-up on HRQoL, we will also be able to identify subgroups with time trends of increasing and decreasing HRQoL levels over time.

Important study limitations of the current study are the differences at baseline between the UHF and MHF group, and the currently limited available follow-up on late effects. Strengths of the study are the cohort setting with a clinical representative study population, the collection of identical information in both a MHF and UHF treatment group, the calculation of MCIDs, and associations with general HRQoL aspects. Further assessments of toxicity risks, such as stenosis and late rectal bleeding, is needed for a comprehensive evaluation of UHF and MHF in our study.

In conclusion, we evaluated HRQoL changes after MHF or UHF in a real-world clinical patient population. In general, levels were comparable with previous reports from clinical HF trials. Clinically relevant deteriorations in bowel and urinary function in the first six months after MHF or UHF were frequent and impacted general QoL aspects. Further research is planned with extended follow-up to better understand the long-term effects and predictors for late toxicity, to enhance optimal treatment choices for PCa patients.

## CRediT authorship contribution statement

**W.D. Heemsbergen:** Methodology, Formal analysis, Visualization, Writing – original draft, Writing – review & editing, Conceptualization, Investigation, Validation, Supervision. **F. Sinzabakira:** Methodology, Formal analysis, Visualization, Writing – original draft. **K.C. de Vries:** Conceptualization, Resources, Writing – review & editing. **M. Franckena:** Conceptualization, Resources, Writing – review & editing. **M.E.M.C. Christianen:** Conceptualization, Resources, Writing – review & editing. **F.E. Froklage:** Resources, Writing – review & editing. **H. Westerveld:** Resources, Writing – review & editing. **L. Incrocci:** Conceptualization, Resources, Supervision, Writing – review & editing.

## Declaration of Competing Interest

The authors declare that they have no known competing financial interests or personal relationships that could have appeared to influence the work reported in this paper.
