# Supplementary material for: Post-treatment changes in bowel and urinary function in prostate cancer patients treated with moderate or ultra-hypofractionation: A prospective cohort study
Source: Clin Transl Radiat Oncol. 2025 Apr 7;53:100955. doi: 10.1016/j.ctro.2025.100955 (PMC12005851; doi:10.1016/j.ctro.2025.100955)

SUPPLEMENTARY FILES

Manuscript: Post-treatment changes in bowel and urinary function in prostate cancer patients treated with moderate or ultra-hypofractionation: a prospective cohort study. Heemsbergen WD et al.

Supplementary Table S1. Mean/median EPIC scores and differences with corresponding p values.

EPIC subdomain	MHF (N=140)	UHF (N=138)	p value MHF vs UHF
Baseline			
Bowel function	92.9/96.4	94.4/96.4	0.3
Bowel bother	95.3/100	97.7/100	<0.01
Urinary function	89.9/93.4	94.5/100	<0.01
Urinary bother	87.2/92.8	93.1/100	<0.01
Urinary incontinence	88.2/100	94.7/100	<0.01
Irritative-obstructive	89.6/92.9	93.5/96.4	<0.01
Month 6			
Bowel function	89.3/92.9	89.7/92.9	1.0
Bowel bother	92.2/98.2	93.7/100	0.09
Urinary function	89.4/93.4	95.2/100	<0.01
Urinary bother	88.6/92.9	93.9/100	<0.01
Urinary incontinence	85.7/100	94.2/100	<0.01
Irritative-obstructive	91.8/96.4	95.3/100	<0.01
M6-Baseline			
Bowel function	-3.4/-3.6	-4.5/-3.6	0.4
Bowel bother	-3.1/0.0	-4.0/0.0	0.5
Urinary function	-0.1/0.0	+0.7/0.0	0.6
Urinary bother	+1.2/0.0	+0.8/0.0	0.8
Urinary incontinence	-2.2/0.0	-0.4/0.0	0.3
Irritative-obstructive	+2.2/0.0	+1.8/0.0	0.8

Abbreviations: MHF=moderate hypofractionation; UHF=ultra hypofractionation.

Supplementary Table S2. Distribution of MCID changes (Month 6 - Baseline) for EPIC urinary domains, evaluated for patients with a baseline urinary function score of 90-100%.

EPIC subdomain	MHF (N=86)	UHF (N=114)	p value MHF vs UHF
**Urinary function**			
No MCID	69.8%	87.7%	0.07
Improved (≥+8%)	5.8%	1.8%	
Worsened (≤ -8%)	24.4%	10.5%	
**Urinary bother**			
No MCID	72.1%	77.2%	0.4
Improved (≥+8%)	8.1%	9.6%	
Worsened (≤ -8%)	19.8%	13.2%	
**Urinary incontinence**			
No MCID	64.0%	79.8%	0.04
Improved (≥+8%)	4.7%	2.6%	
Worsened (≤ -8%)	31.4%	17.5%	
**Urinary irritative -obstructive**			
No MCID	72.1%	81.6%	0.2
Improved (≥+8%)	20.6%	17.4%	
Worsened (≤ -8%)	19.1%	8.0%	

Abbreviations: MCID=minimal clinically important differences; MHF=moderate hypofractionation; UHF=ultra hypofractionation.

Supplementary Table S3. Univariable logistic regression models for the endpoints of MCID deteriorations in Health VAS score (Odds Ratios with 95% confidence intervals). Significant results are in bold.

	**VAS Health MCID ≤-8% OR (95% CI)**
Baseline characteristics	
T3 vs T1-2	2.19 (1.2-4.0)
ADT yes vs no	**1.71** (1.0-2.9)
MHF vs UHF	0.89 (0.5-1.5)
Age ≥75 vs <75	0.86 (0.5-1.5)
CCI score ≥2	0.97 (0.6-1.7)
Diabetes	1.24 (0.6-1.4)
Cardiovascular history	1.25 (0.7-2.2)
Abdominal surgery	1.09 (0.6-1.9)
LUTS medication	0.66 (0.4-1.2)
Baseline scores	
VAS health <80 vs ≥80	0.67 (0.4-1.2)
Bowel function <90 vs ≥90	0.81 (0.4-1.5)
Urinary function <90 vs ≥90	0.91 (0.4-2.0)
Acute toxicity	
Acute grade ≥2 Bowel	0.91 (0.5-1.6)
Acute grade ≥2 Urinary	1.43 (0.8-2.5)

Abbreviations: ADT=androgen deprivation therapy; CCI=Charlson Comorbidity Index; CI=confidence interval; MHF=moderate hypofractionation; LUTS=lower urinary tract symptoms; MCID=minimal clinically important differences; OR=odds ratio; UHF=ultra hypofractionation; VAS=visual analogue scale.

Supplementary Table S4. Associations between changes in M6-Baseline EQ-5D-5L dimensions and M6-Baseline EPIC subdomain scores (Spearman rank correlations: correlation coefficient and p value). Positive correlations indicate that worse scores correlate with worse scores / improved scores correlates with improved scores. Negative correlations indicate that function worsening in one scale correlates with improvement in function on the other scale. Significant results in bold.

**EPIC**<br>**EQ-5D-5L**	**Bowel function**	**Bowel bother**	**Urinary function**	**Urinary bother**	**Urinary irritative**	**Urinary incontinence**
**Mobility**	0.02, p=0.7	0.08, p=0.2	0.10, p=0.12	0.02, p=0.8	0.01, p=0.9	0.10, p=0.3
**Self-care**	0.07, p=0.3	0.08, p=0.2	0.02, p=0.7	0.01, p=0.9	0.01, p=0.9	0.05, p=0.4
**Usual activities**	**0.23, p<0.001**	**0.20, p<0.001**	0.11, p=0.07	**0.17, p=0.005**	**0.13, p=0.04**	0.12, p=0.06
**Pain/discomfort**	**0.13, p=0.03**	0.10, p=0.08	0.08, p=0.2	**0.13, p=0.036**	**0.12, p=0.047**	0.02, p=0.7
**Anxiety/depression**	**0.18, p=0.003**	**0.18, p=0.002**	0.12, p=0.06	**0.14, p=0.02**	**0.14, p=0.02**	**0.13, p=0.04**

Supplementary Figure S1. Upper panes: distribution of baseline scores for the EPIC domains bowel function, urinary function, and for the EQ-5D-5L VAS score on general health perception (N=278). **Lower panes**: distribution of changes in score between baseline and M6 for bowel function, urinary function, and VAS score general health perception.

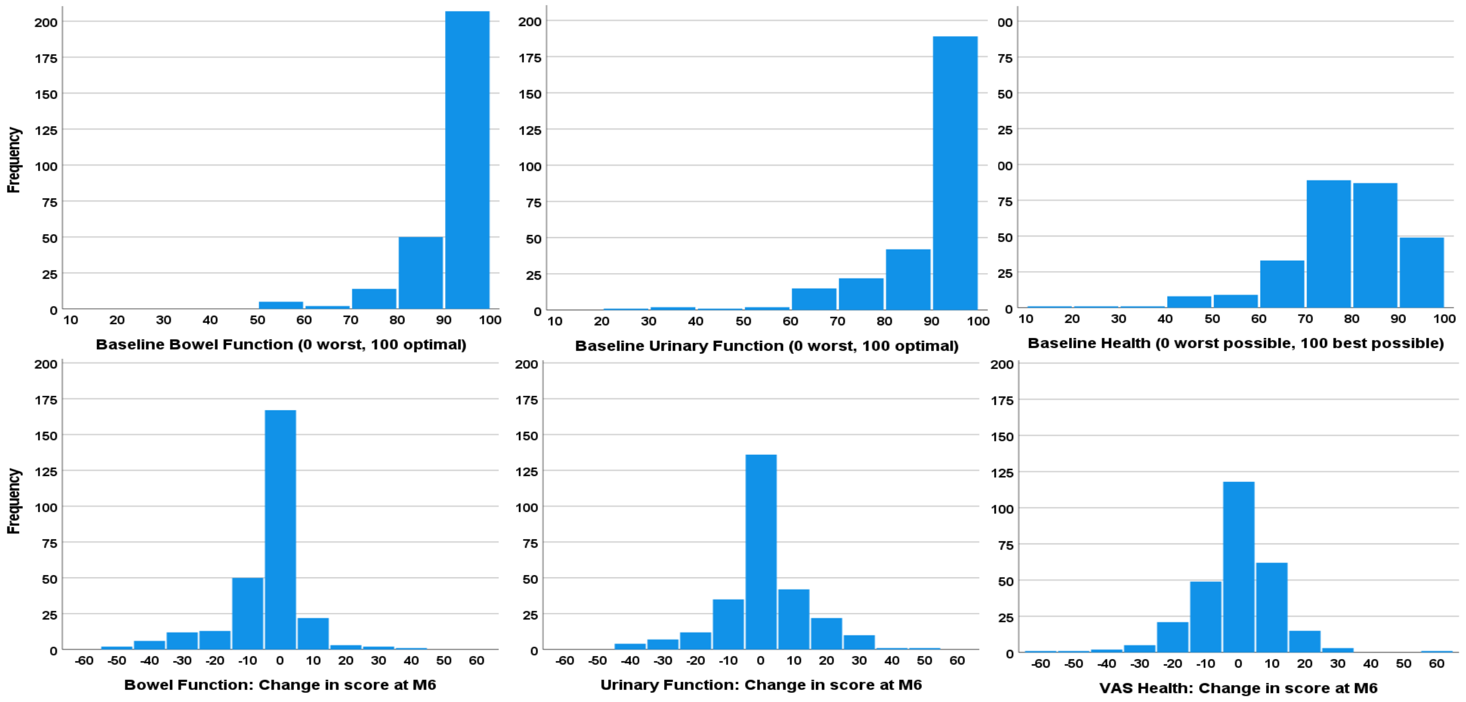

Supplementary Figure S2. Histogram of changes in VAS health perception score for the moderate hypofractionation (MHF) and ultra-hypofractionation (UHF) patient group**.**

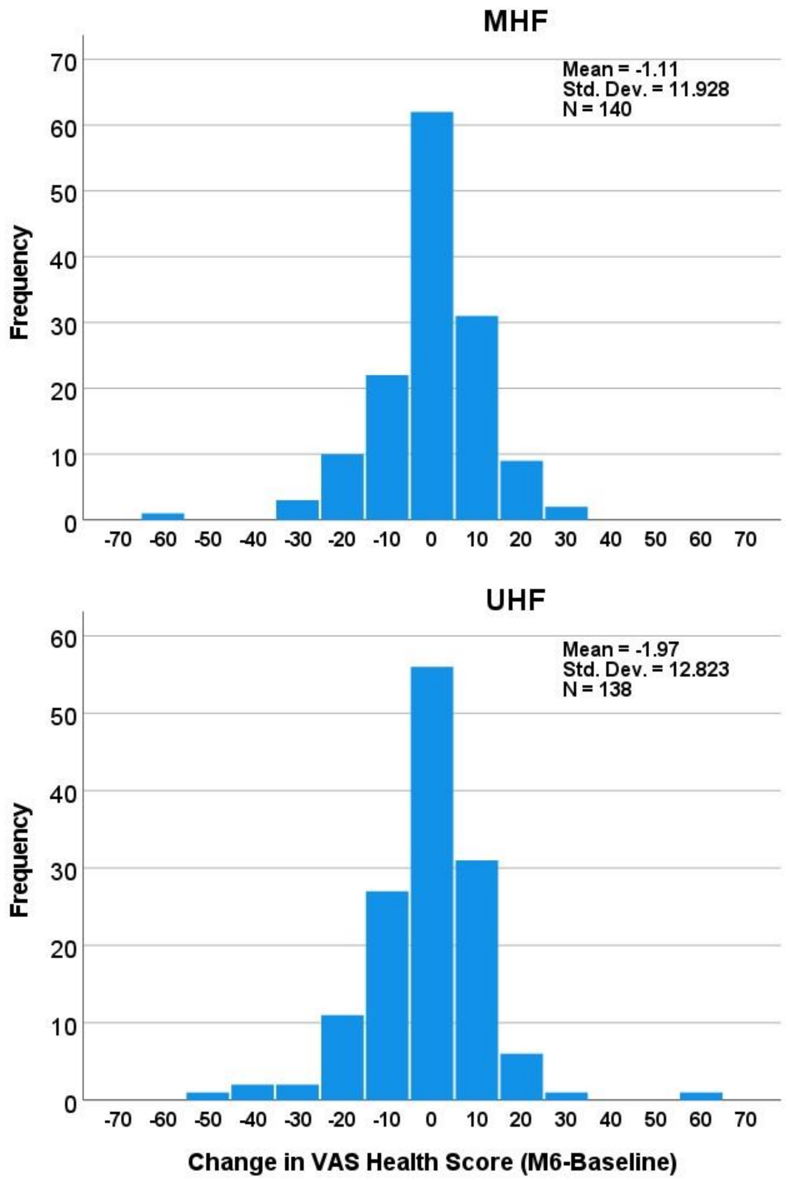

Supplementary Figure S3. Reported average scores for bother of symptoms at Month 6 post-treatment (bowel and urinary), stratified for patients with and without MCID (minimally clinical important difference) <-8% (deterioration).

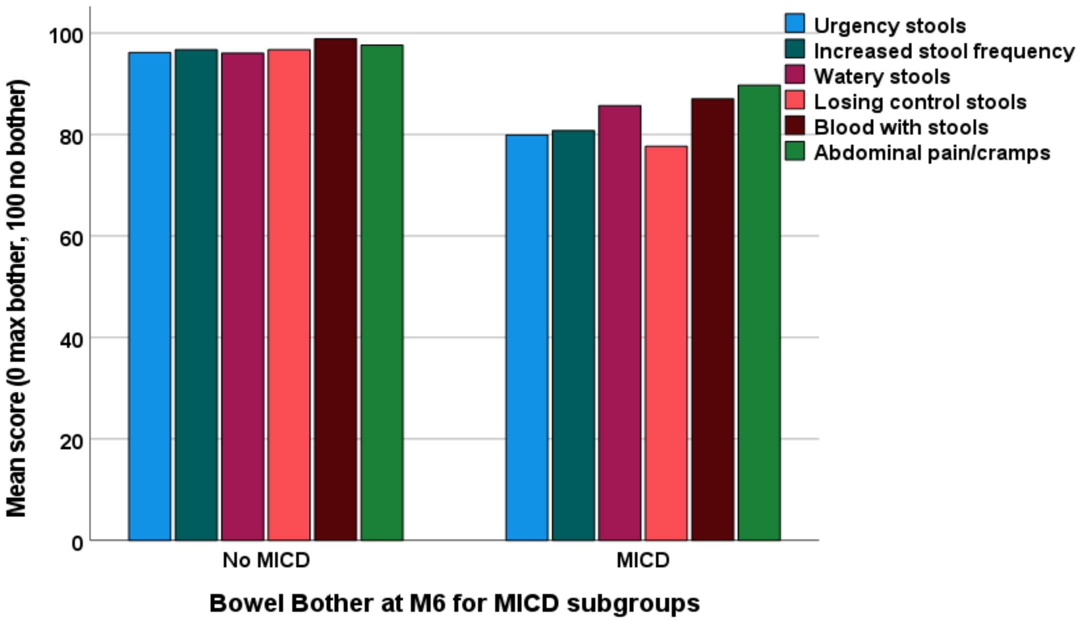

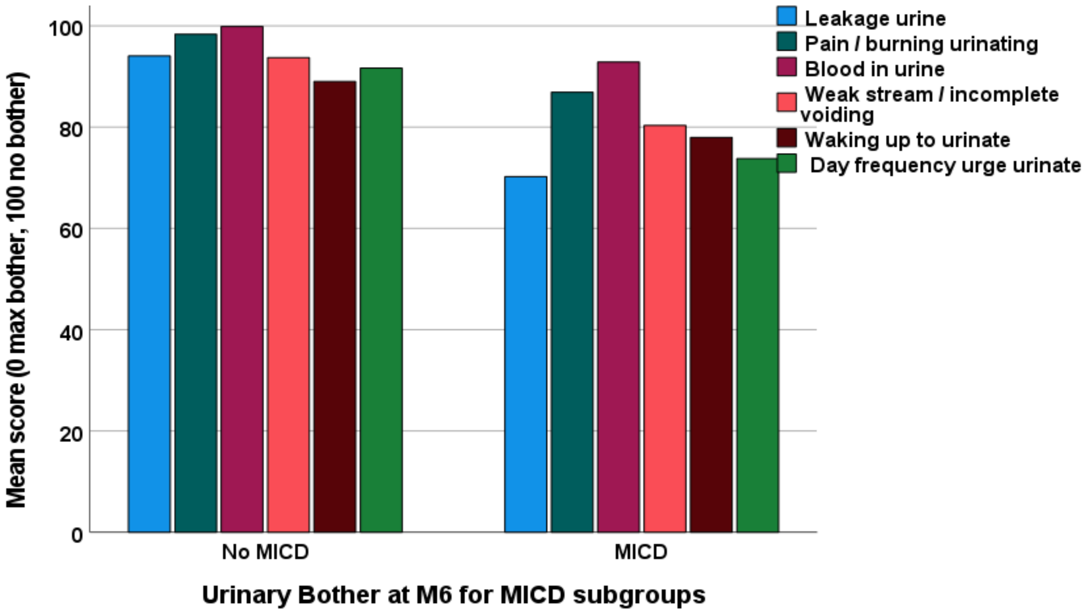

Supplement: Supplementary Data 1 [file mmc1.pdf]
